# Crystal structure of strontium thio­sulfate monohydrate

**DOI:** 10.1107/S2056989020000353

**Published:** 2020-01-17

**Authors:** Wilhelm Klein

**Affiliations:** a Technische Universität München, Department of Chemistry, Lichtenbergstr. 4, 85747 Garching, Germany

**Keywords:** crystal structure, thio­sulfate, strontium, hydrate, hydrogen bonding

## Abstract

SrS_2_O_3_·H_2_O was obtained from an aqueous solution of Na_2_S_2_O_3_ and Sr(NO_3_)_2_ and crystallizes in space group *P*  with all atoms at general positions. The Sr^2+^ ion exhibits an [8 + 1] coordination defined by two terminal S and six O atoms of thio­sulfate ions, one of the latter at a longer distance, and by one O atom of a water mol­ecule. Two thio­sulfate anions act as bidentate, four as monodentate ligands. The structure consists of mainly ionically inter­acting layers lying parallel to the crystallographic *ab* plane. The layers are connected by O—H⋯S and O—H⋯O hydrogen bonds of moderate strength.

## Abstract 2

The title compound represents one of the hydrous alkaline earth thio­sulfates with the lowest amount of crystal water known so far. The structure consists of layers, which are connected by weak hydrogen bonds.

## Chemical context   

Although thio­sulfuric acid and its salts are common topics in textbooks of inorganic chemistry, the preparation of the pure acid was achieved just recently by a sophisticated synthesis *via* reaction of Na_2_S_2_O_3_ and anhydrous HF (Hopfinger *et al.*, 2018 ▸). Its salts are much better explored, as they are naturally and geologically widely spread (Caufield & Raiswell, 1999 ▸), and Na_2_S_2_O_3_, as well as (NH_4_)_2_S_2_O_3_, is produced on a large industrial scale (Barberá *et al.*, 2012 ▸). To date, thio­sulfates of alkaline earth metals are solely known as hydrates. For example, MgS_2_O_3_·6H_2_O has been investigated by Elerman *et al.* (1983 ▸) to determine its deformation electron density, and the first example of an S—H hydrogen bond that was confirmed by a single crystal-structure determination was found in BaS_2_O_3_·H_2_O (Manojlović-Muir, 1969 ▸).

Next to SrS_2_O_3_·5H_2_O (Held & Bohatý, 2004 ▸), the title compound represents the second known crystal structure of a hydrate of strontium thio­sulfate. As one route of preparation, the penta­hydrate has been crystallized from aqueous solutions of Na_2_S_2_O_3_ and Sr(NO_3_)_2_, whereby these solutions were reported to show a tendency to decompose, inhibiting the growth of larger single crystals (Held & Bohatý, 2004 ▸). A possible step within the decomposition process, and maybe a competing product in a later stage of crystallization, might be associated with the monohydrate, the crystal structure of which is presented here.

## Structural commentary   

SrS_2_O_3_·H_2_O crystallizes in the space group *P* 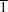 with one formula unit in the asymmetric unit and all atoms on general positions. Many structural features resemble the closely related penta­hydrate of SrS_2_O_3_. The thio­sulfate anion adopts a slightly distorted tetra­hedral shape with a mean bond angle of 109.47° where the average O—S—O angles (110.32°) are slightly larger than the S—S—O angles (108.62°). Similar to the S—S bond length found in the penta­hydrate (1.995 Å), the S—S bond length of 2.0044 (7) Å in the monohydrate is between those of the Ca (2.008 Å) and the Ba (1.979 Å) salts. The S—O bond lengths are between 1.466 (2) Å and 1.478 (2) Å and are in the same range as those of other alkaline earth thio­sulfate hydrates (Table 1 ▸).

**Figure 1:**
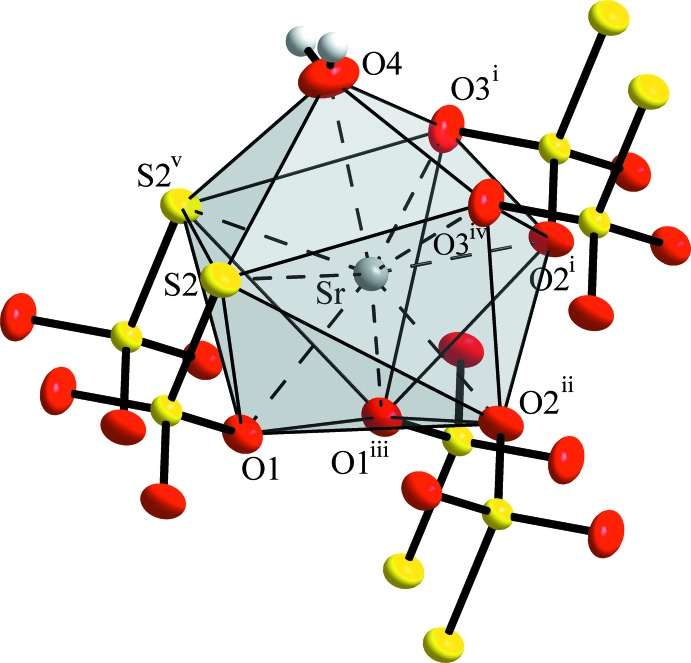
Coordination polyhedron of the Sr^2+^ cation in SrS_2_O_3_·H_2_O. Anisotropic displacement ellipsoids are drawn at the 70% probability level; H atoms are shown with arbitrary radius. [Symmetry codes: (i) *x* − 1, *y* + 1, *z*; (ii) −*x* + 1, −*y* + 1, −*z*; (iii) −*x*, −*y* + 1, −*z*; (iv) *x*, *y* + 1, *z*; (v) *x* − 1, *y*, *z*.]

The Sr^2+^ cation is coordinated by five O and two S atoms, belonging to six neighbouring S_2_O_3_^2–^ anions, and one additional O atom of an H_2_O mol­ecule. One of the anions acts as a bidentate S/O ligand, while the remaining five coordinate only *via* one S or O atom, respectively. These six O ligands are found in narrow Sr—O distances ranging from 2.531 (2) to 2.623 (2) Å, whereas the S atoms exhibit Sr—S distances of 3.1618 (6) and 3.2379 (6) Å. A more remote O atom at a distance of 3.305 (2) Å might also be ascribed to the first coordination sphere, although exhibiting a larger distance than the neighbouring S atoms. This [8 + 1] coordination of the Sr^2+^ atom (Fig. 1 ▸) again resembles the ninefold coordination of the cation in SrS_2_O_3_·5H_2_O, with the difference being that in the penta­hydrate no S atoms are found in the first coordination sphere of Sr^2+^, but four water mol­ecules instead. As a consequence of the presence of the larger S atoms close to Sr^2+^, in the title structure one O atom is shifted into an outer region of the coordination shell and thus is found at a considerably longer distance.

**Figure 2:**
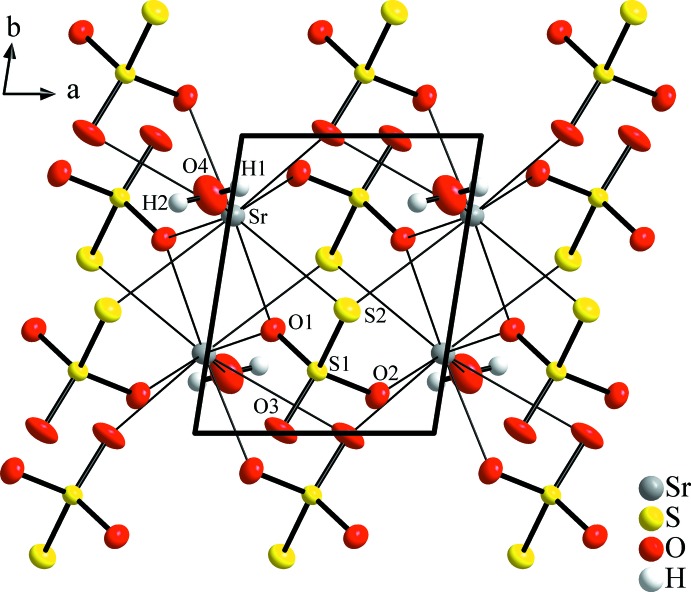
Crystal structure of SrS_2_O_3_·H_2_O, in a view onto (001). Displacement ellipsoids are shown as in Fig. 1 ▸.

**Figure 3:**
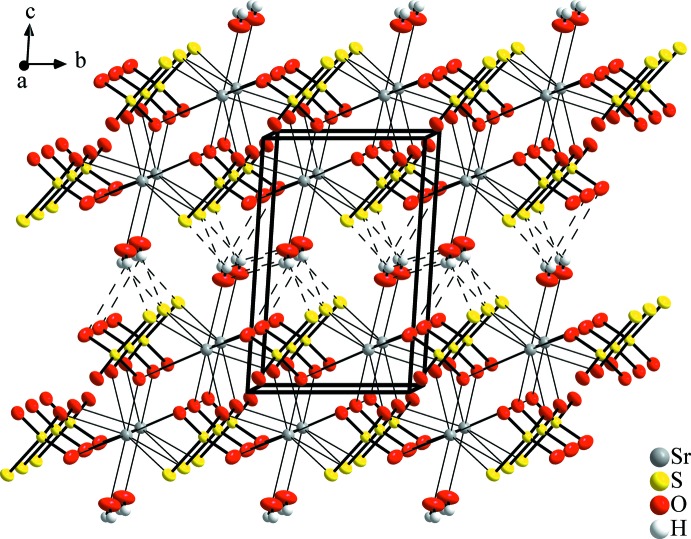
A projection of the crystal structure of SrS_2_O_3_·H_2_O, approximately along [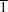
00]. Displacement ellipsoids are shown as in Fig. 1 ▸.

As a characteristic feature of the crystal structure, SrS_2_O_3_·H_2_O is made up from layers extending parallel to the crystallographic *ab* plane (Fig. 2 ▸). Within the layers, the condensed coordination polyhedra are packed alternately to form double sheets in such a way that the terminal S2 atoms and water mol­ecules are directed towards the layer boundaries (Fig. 3 ▸). The layers are linked by hydrogen bonds of medium strength between the water mol­ecules (O4⋯O4^ii^) and the water mol­ecules and thio­sulfate anions *via* S2 atoms (Table 2 ▸). This involves also a bifurcated hydrogen bond O4—H1⋯(O4^ii^/S2^iii^). The O4—H2⋯S2^i^ bond as well as the *D*⋯*A* distances are in the same range as in SrS_2_O_3_·5H_2_O (Held & Bohatý, 2004 ▸). For the H1 atom, a disorder model similar to that proposed for BaS_2_O_3_·H_2_O (Manojlović-Muir, 1975 ▸) was considered, which would result in shorter and more linear O4—H1⋯O4^ii^ and O4—H1⋯S2^iii^ bonds. However, a reasonable refinement of these disordered H atoms was not possible.

**Figure 4:**
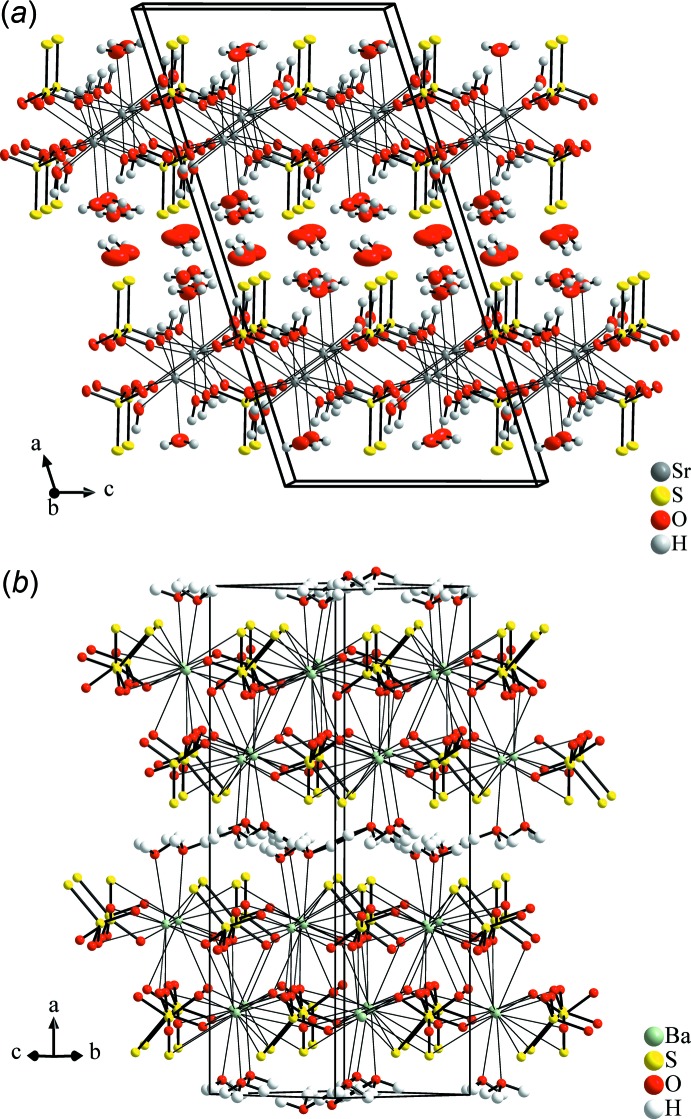
Illustration of the structural relationship between the title compound, SrS_2_O_3_·H_2_O, and (*a*) SrS_2_O_3_·5H_2_O (view approximately along [0
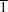
0]) and (*b*) BaS_2_O_3_·H_2_O (view approximately along [011]).

A striking analogy to the packing of the penta­hydrate structure (Fig. 4 ▸ *a*) is apparent. With the presence of five water mol­ecules instead of one, the main packing of ions in SrS_2_O_3_·5H_2_O is only slightly changed as a result of the coordination of additional water mol­ecules to the Sr^2+^ cation and widened by two non-coordinating and hydrogen-bonded water mol­ecules situated between the layers. The S—S bond is nearly orthogonal to the layer plane; however, the layer boundaries are also formed by S atoms and water mol­ecules, both forming hydrogen bonds. The very close relationship between the two crystal structures suggests a topotactical degradation of the penta­hydrate. Because both hydrates were crystallized at room temperature, a temperature dependence of the crystallization does not seem to be the only possible driving force. An ageing process triggered by concentration or thermodynamic stability must be taken into account as well. The degradation process, possibly running *via* another so far unknown trihydrate after removal of the free water mol­ecules, was not investigated up to now, and in addition a thermal analysis of the title compound could not been carried out because of the presence of large amounts of indistinguishable crystalline by-products, *viz*. NaNO_3_ and Sr(NO_3_)_2_.

The ortho­rhom­bic structure of BaS_2_O_3_·H_2_O is likewise found to form layers, which are separated by water mol­ecules (Fig. 4 ▸ *b*; Nardelli & Fava, 1962 ▸; Manojlović-Muir, 1975 ▸). Similar to the Sr homologue, two terminal S atoms are part of the first coordination sphere of the Ba^2+^ cation which has, caused by the larger ion radius, a different environment, namely by five thio­sulfate anions as bidentate ligands and one additional water mol­ecule. Inter­estingly, while the number of atoms forming the first coordination sphere is higher in the Ba compound, the number of directly coordinating anions is smaller.

## Database survey   

Besides SrS_2_O_3_, crystal structure determinations for three further alkaline-earth thio­sulfates have been reported, all of them as hydrates: BaS_2_O_3_·H_2_O (Nardelli & Fava, 1962 ▸; Manojlović-Muir, 1975 ▸), CaS_2_O_3_·6H_2_O (Held & Bohatý, 2004 ▸), and MgS_2_O_3_·6H_2_O (Nardelli *et al.*, 1962 ▸; Baggio *et al.*, 1969 ▸; Elerman *et al.*, 1982 ▸,1983 ▸). Together with the known Sr compounds, SrS_2_O_3_·5H_2_O (Held & Bohatý, 2004 ▸) and the new monohydrate, the trend of incorporating smaller amounts of water into stable crystal structures with increasing cation radius is obvious for alkaline-earth metal thio­sulfates. With the exception of two of the five water mol­ecules in SrS_2_O_3_·5H_2_O, all water mol­ecules in these compounds coordinate to the divalent cations. This trend is confirmed by divalent transition-metal thio­sulfates, as there are those of Ni as the hexa­hydrate (Elerman *et al.*, 1978 ▸; isostructural with the Mg salt) and of Cd as the dihydrate (Baggio *et al.*, 1997 ▸). The only crystal structure of a hydrate-free thio­sulfate of a divalent cation is reported for Pb (Christensen *et al.*, 1991 ▸). Table 1 ▸ collates S—S and averaged S—O bond lengths in the corresponding structures of these thio­sulfates.

## Synthesis and crystallization   

Crystals of SrS_2_O_3_·H_2_O were grown from an aqueous solution of Na_2_S_2_O_3_·5H_2_O and Sr(NO_3_)_2_. The solution was stored at room temperature and the solvent was evaporated very slowly over several months. Single crystals were isolated from highly concentrated solutions where only a little of the mother liquor remained. Besides the title compound, crystals of NaNO_3_ and surplus Sr(NO_3_)_2_ were also found, and all of these compounds were identified in the X-ray powder pattern of the reaction mixture after drying at room temperature. From all these experiments, no hints of the presence of the penta­hydrate were found.

## Refinement   

Crystal data, data collection and structure refinement details are summarized in Table 3 ▸. Hydrogen atoms were refined with a restrained O—H distance of 0.85 (5) Å and *U*_iso_(H) = 1.5*U*_eq_(O). A free refinement of H-atom positions resulted in a reliable shape for the water mol­ecule and orientation with respect to possible hydrogen bonds, but included one short O—H distance of only 0.5 Å.

## Supplementary Material

Crystal structure: contains datablock(s) global, I. DOI: 10.1107/S2056989020000353/wm5535sup1.cif

Structure factors: contains datablock(s) I. DOI: 10.1107/S2056989020000353/wm5535Isup2.hkl

CCDC reference: 1977322

Additional supporting information:  crystallographic information; 3D view; checkCIF report

## Figures and Tables

**Table 1:** S—S and averaged S—O bond lengths (Å) in thio­sulfates of divalent cations

Cation/solvent mol­ecules	S—S	mean S—O
Ba^2+^/1 H_2_O^*a*^	1.979	1.477
Sr^2+^/5 H_2_O^*b*^	1.995	1.472
Sr^2+^/1 H_2_O^*c*^	2.004	1.474
Ca^2+^/6 H_2_O^*b*^	2.008	1.468
Mg^2+^/6 H_2_O^*d*^	2.019	1.471
Ni^2+^/6 H_2_O^*e*^	2.015	1.459
Cd^2+^/2 H_2_O^*f*^	2.056	1.454
Pb^2+^/0 H_2_O^*g*^	2.11	1.455

Notes: (*a*) Manojlović-Muir (1975 ▸); (*b*) Held & Bohatý (2004 ▸); (*c*) this work; (*d*) Elerman *et al.* (1983 ▸); (*e*) Elerman *et al.* (1978 ▸); (*f*) Baggio *et al.* (1997 ▸); (*g*) Christensen *et al.* (1991 ▸).

**Table 2:** Hydrogen-bond geometry (Å, °)

*D*—H⋯*A*	*D*—H	H⋯*A*	*D*⋯*A*	*D*—H⋯*A*
O4—H2⋯S2^i^	0.76 (3)	2.62 (4)	3.344 (2)	163 (4)
O4—H1⋯O4^ii^	0.78 (4)	2.42 (4)	2.962 (4)	128 (4)
O4—H1⋯S2^iii^	0.78 (4)	2.84 (4)	3.458 (2)	138 (4)

Symmetry codes: (i) 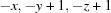; (ii) 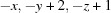; (iii) 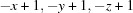.

**Table 3:** Experimental details

Crystal data	
Chemical formula	SrS_2_O_3_·H_2_O
*M*_r_	217.76
Crystal system, space group	Triclinic, *P* 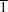
Temperature (K)	297
*a*, *b*, *c* (Å)	4.6858 (2), 5.9178 (3), 9.0167 (4)
α, β, γ (°)	84.889 (2), 87.284 (2), 80.785 (2)
*V* (Å^3^)	245.68 (2)
*Z*	2
Radiation type	Mo *K*α
μ (mm^−1^)	11.72
Crystal size (mm)	0.42 × 0.30 × 0.16

Data collection	
Diffractometer	Bruker APEXII CCD
Absorption correction	Numerical (*SADABS*; Krause *et al.*, 2015 ▸)
*T*_min_, *T*_max_	0.037, 0.264
No. of measured, independent and observed [*I* > 2σ(*I*)] reflections	8011, 1504, 1481
*R*_int_	0.054
(sin θ/λ)_max_ (Å^−1^)	0.715

Refinement	
*R*[*F* ^2^ > 2σ(*F* ^2^)], *wR*(*F* ^2^), *S*	0.024, 0.061, 1.10
No. of reflections	1504
No. of parameters	71
No. of restraints	2
H-atom treatment	Only H-atom coordinates refined
Δρ_max_, Δρ_min_ (e Å^−3^)	0.97, −0.86

Computer programs: *APEX3* and *SAINT* (Bruker, 2015 ▸), *SHELXS97* (Sheldrick, 2008 ▸), *SHELXL2014* (Sheldrick, 2015 ▸), *DIAMOND* (Brandenburg & Putz, 2012 ▸) and *publCIF* (Westrip, 2010 ▸).

## supplementary crystallographic information

### Crystal data

**Table:**

SrS_2_O_3_·H_2_O	*Z* = 2
*M**_r_* = 217.76	*F*(000) = 208
Triclinic, *P*1	*D*_x_ = 2.944 Mg m^−^^3^
*a* = 4.6858 (2) Å	Mo *K*α radiation, λ = 0.71073 Å
*b* = 5.9178 (3) Å	Cell parameters from 7034 reflections
*c* = 9.0167 (4) Å	θ = 2.3–30.6°
α = 84.889 (2)°	µ = 11.72 mm^−^^1^
β = 87.284 (2)°	*T* = 297 K
γ = 80.785 (2)°	Block, colourless
*V* = 245.68 (2) Å^3^	0.42 × 0.30 × 0.16 mm

### Data collection

**Table:**

Bruker APEXII CCD diffractometer	1504 independent reflections
Radiation source: rotating anode FR591	1481 reflections with *I* > 2σ(*I*)
MONTEL optic monochromator	*R*_int_ = 0.054
Detector resolution: 16 pixels mm^-1^	θ_max_ = 30.6°, θ_min_ = 2.3°
φ– and ω–rotation scans	*h* = −6→6
Absorption correction: numerical (*SADABS*; Krause *et al.*, 2015)	*k* = −8→8
*T*_min_ = 0.037, *T*_max_ = 0.264	*l* = −12→12
8011 measured reflections	

### Refinement

**Table:**

Refinement on *F*^2^	Secondary atom site location: difference Fourier map
Least-squares matrix: full	Hydrogen site location: difference Fourier map
*R*[*F*^2^ > 2σ(*F*^2^)] = 0.024	Only H-atom coordinates refined
*wR*(*F*^2^) = 0.061	*w* = 1/[σ^2^(*F*_o_^2^) + (0.0324*P*)^2^ + 0.2036*P*] where *P* = (*F*_o_^2^ + 2*F*_c_^2^)/3
*S* = 1.10	(Δ/σ)_max_ = 0.001
1504 reflections	Δρ_max_ = 0.97 e Å^−^^3^
71 parameters	Δρ_min_ = −0.86 e Å^−^^3^
2 restraints	Extinction correction: SHELXL2014 (Sheldrick, 2015), Fc^*^=kFc[1+0.001xFc^2^λ^3^/sin(2θ)]^-1/4^
Primary atom site location: structure-invariant direct methods	Extinction coefficient: 0.085 (5)

### Special details

**Table:**

Geometry. All esds (except the esd in the dihedral angle between two l.s. planes) are estimated using the full covariance matrix. The cell esds are taken into account individually in the estimation of esds in distances, angles and torsion angles; correlations between esds in cell parameters are only used when they are defined by crystal symmetry. An approximate (isotropic) treatment of cell esds is used for estimating esds involving l.s. planes.

### Fractional atomic coordinates and isotropic or equivalent isotropic displacement parameters (Å^2^)

**Table:**

	*x*	*y*	*z*	*U*_iso_*/*U*_eq_	
Sr	0.01493 (4)	0.73223 (3)	0.16811 (2)	0.01372 (10)	
S1	0.47043 (11)	0.20793 (8)	0.16097 (5)	0.01113 (12)	
S2	0.55500 (12)	0.41404 (9)	0.31260 (6)	0.01678 (13)	
O1	0.2582 (3)	0.3477 (3)	0.06042 (17)	0.0163 (3)	
O2	0.7385 (3)	0.1265 (3)	0.07573 (19)	0.0178 (3)	
O3	0.3580 (4)	0.0090 (3)	0.23659 (19)	0.0221 (3)	
O4	−0.0933 (5)	0.7998 (4)	0.4397 (2)	0.0309 (4)	
H1	0.024 (9)	0.827 (8)	0.491 (5)	0.046*	
H2	−0.223 (8)	0.774 (7)	0.487 (4)	0.046*	

### Atomic displacement parameters (Å^2^)

**Table:**

	*U*^11^	*U*^22^	*U*^33^	*U*^12^	*U*^13^	*U*^23^
Sr	0.01592 (13)	0.01292 (12)	0.01266 (13)	−0.00269 (7)	−0.00130 (7)	−0.00167 (7)
S1	0.0116 (2)	0.0105 (2)	0.0117 (2)	−0.00225 (16)	−0.00057 (16)	−0.00182 (16)
S2	0.0202 (3)	0.0170 (2)	0.0145 (2)	−0.00505 (19)	−0.00235 (19)	−0.00465 (18)
O1	0.0159 (7)	0.0160 (7)	0.0168 (7)	0.0000 (6)	−0.0053 (6)	−0.0030 (6)
O2	0.0145 (7)	0.0194 (7)	0.0188 (7)	0.0006 (6)	0.0027 (6)	−0.0057 (6)
O3	0.0316 (9)	0.0187 (7)	0.0191 (8)	−0.0146 (7)	−0.0033 (7)	0.0023 (6)
O4	0.0339 (11)	0.0433 (11)	0.0187 (9)	−0.0151 (9)	0.0037 (8)	−0.0071 (8)

### Geometric parameters (Å, º)

**Table:**

Sr—O4	2.531 (2)	S1—O2	1.4768 (16)
Sr—O2^i^	2.5698 (16)	S1—O1	1.4776 (15)
Sr—O2^ii^	2.5708 (17)	S1—S2	2.0044 (7)
Sr—O1^iii^	2.5934 (15)	S1—Sr^vii^	3.4816 (5)
Sr—O3^iv^	2.6010 (17)	S2—Sr^viii^	3.2379 (6)
Sr—O1	2.6226 (15)	O1—Sr^iii^	2.5934 (15)
Sr—S2	3.1618 (6)	O2—Sr^vii^	2.5698 (16)
Sr—S2^v^	3.2379 (6)	O2—Sr^ii^	2.5708 (16)
Sr—O3^i^	3.305 (2)	O3—Sr^ix^	2.6010 (17)
Sr—S1	3.4768 (5)	O3—Sr^vii^	3.305 (2)
Sr—S1^i^	3.4816 (5)	O4—H1	0.78 (4)
Sr—Sr^vi^	4.1762 (4)	O4—H2	0.76 (3)
S1—O3	1.4663 (16)		
			
O4—Sr—O2^i^	93.29 (7)	O1^iii^—Sr—S1^i^	80.94 (3)
O4—Sr—O2^ii^	145.14 (6)	O3^iv^—Sr—S1^i^	86.26 (4)
O2^i^—Sr—O2^ii^	71.34 (6)	O1—Sr—S1^i^	150.22 (3)
O4—Sr—O1^iii^	138.36 (6)	S2—Sr—S1^i^	152.443 (14)
O2^i^—Sr—O1^iii^	75.45 (5)	S2^v^—Sr—S1^i^	89.500 (14)
O2^ii^—Sr—O1^iii^	69.30 (5)	O3^i^—Sr—S1^i^	24.78 (3)
O4—Sr—O3^iv^	73.47 (6)	S1—Sr—S1^i^	170.625 (18)
O2^i^—Sr—O3^iv^	77.99 (6)	O4—Sr—Sr^vi^	122.71 (6)
O2^ii^—Sr—O3^iv^	72.79 (5)	O2^i^—Sr—Sr^vi^	35.68 (4)
O1^iii^—Sr—O3^iv^	138.90 (5)	O2^ii^—Sr—Sr^vi^	35.66 (4)
O4—Sr—O1	126.88 (7)	O1^iii^—Sr—Sr^vi^	68.15 (3)
O2^i^—Sr—O1	139.15 (5)	O3^iv^—Sr—Sr^vi^	71.93 (4)
O2^ii^—Sr—O1	77.29 (5)	O1—Sr—Sr^vi^	109.27 (4)
O1^iii^—Sr—O1	69.30 (5)	S2—Sr—Sr^vi^	129.735 (12)
O3^iv^—Sr—O1	116.91 (6)	S2^v^—Sr—Sr^vi^	134.574 (12)
O4—Sr—S2	80.16 (6)	O3^i^—Sr—Sr^vi^	80.32 (3)
O2^i^—Sr—S2	152.24 (4)	S1—Sr—Sr^vi^	124.721 (11)
O2^ii^—Sr—S2	98.85 (4)	S1^i^—Sr—Sr^vi^	58.002 (9)
O1^iii^—Sr—S2	126.47 (3)	O3—S1—O2	108.96 (11)
O3^iv^—Sr—S2	74.28 (4)	O3—S1—O1	112.09 (10)
O1—Sr—S2	57.20 (3)	O2—S1—O1	109.91 (9)
O4—Sr—S2^v^	69.28 (5)	O3—S1—S2	109.49 (7)
O2^i^—Sr—S2^v^	108.83 (4)	O2—S1—S2	109.77 (7)
O2^ii^—Sr—S2^v^	144.82 (4)	O1—S1—S2	106.59 (7)
O1^iii^—Sr—S2^v^	76.67 (4)	O3—S1—Sr	115.97 (8)
O3^iv^—Sr—S2^v^	142.38 (4)	O2—S1—Sr	134.05 (7)
O1—Sr—S2^v^	82.74 (4)	O1—S1—Sr	43.98 (6)
S2—Sr—S2^v^	94.134 (15)	S2—S1—Sr	64.00 (2)
O4—Sr—O3^i^	65.98 (6)	O3—S1—Sr^vii^	70.87 (8)
O2^i^—Sr—O3^i^	46.05 (5)	O2—S1—Sr^vii^	41.53 (7)
O2^ii^—Sr—O3^i^	114.88 (5)	O1—S1—Sr^vii^	141.36 (6)
O1^iii^—Sr—O3^i^	78.38 (4)	S2—S1—Sr^vii^	108.24 (2)
O3^iv^—Sr—O3^i^	104.37 (6)	Sr—S1—Sr^vii^	170.625 (18)
O1—Sr—O3^i^	138.65 (4)	S1—S2—Sr	81.26 (2)
S2—Sr—O3^i^	144.49 (3)	S1—S2—Sr^viii^	109.00 (2)
S2^v^—Sr—O3^i^	64.83 (3)	Sr—S2—Sr^viii^	94.134 (15)
O4—Sr—S1	106.66 (6)	S1—O1—Sr^iii^	134.66 (9)
O2^i^—Sr—S1	159.58 (4)	S1—O1—Sr	112.99 (8)
O2^ii^—Sr—S1	89.34 (4)	Sr^iii^—O1—Sr	110.70 (5)
O1^iii^—Sr—S1	91.84 (3)	S1—O2—Sr^vii^	116.07 (9)
O3^iv^—Sr—S1	103.11 (4)	S1—O2—Sr^ii^	134.93 (10)
O1—Sr—S1	23.03 (3)	Sr^vii^—O2—Sr^ii^	108.66 (6)
S2—Sr—S1	34.737 (12)	S1—O3—Sr^ix^	135.58 (10)
S2^v^—Sr—S1	82.985 (14)	S1—O3—Sr^vii^	84.35 (8)
O3^i^—Sr—S1	147.67 (3)	Sr^ix^—O3—Sr^vii^	104.37 (6)
O4—Sr—S1^i^	75.63 (6)	Sr—O4—H1	122 (3)
O2^i^—Sr—S1^i^	22.40 (4)	Sr—O4—H2	128 (3)
O2^ii^—Sr—S1^i^	93.64 (4)	H1—O4—H2	109 (4)

Symmetry codes: (i) *x*−1, *y*+1, *z*; (ii) −*x*+1, −*y*+1, −*z*; (iii) −*x*, −*y*+1, −*z*; (iv) *x*, *y*+1, *z*; (v) *x*−1, *y*, *z*; (vi) −*x*, −*y*+2, −*z*; (vii) *x*+1, *y*−1, *z*; (viii) *x*+1, *y*, *z*; (ix) *x*, *y*−1, *z*.

### Hydrogen-bond geometry (Å, º)

**Table:**

*D*—H···*A*	*D*—H	H···*A*	*D*···*A*	*D*—H···*A*
O4—H2···S2^x^	0.76 (3)	2.62 (4)	3.344 (2)	163 (4)
O4—H1···O4^xi^	0.78 (4)	2.42 (4)	2.962 (4)	128 (4)
O4—H1···S2^xii^	0.78 (4)	2.84 (4)	3.458 (2)	138 (4)

Symmetry codes: (x) −*x*, −*y*+1, −*z*+1; (xi) −*x*, −*y*+2, −*z*+1; (xii) −*x*+1, −*y*+1, −*z*+1.
